# Lipopolysaccharide-Induced Nitric Oxide, Prostaglandin E2, and Cytokine Production of Mouse and Human Macrophages Are Suppressed by Pheophytin-b

**DOI:** 10.3390/ijms18122637

**Published:** 2017-12-06

**Authors:** Chun-Yu Lin, Wen-Hung Wang, Shin-Huei Chen, Yu-Wei Chang, Ling-Chien Hung, Chung-Yi Chen, Yen-Hsu Chen

**Affiliations:** 1 Division of Infectious Diseases, Department of Internal Medicine, Kaohsiung Medical University Hospital, Kaohsiung 807, Taiwan; infectionman@gmail.com (C.-Y.L.); bole0918@gmail.com (W.-H.W.); lavender99kimo@yahoo.com.tw (L.-C.H.); 2 Sepsis Research Center, Graduate Institute of Medicine, College of Medicine, Kaohsiung Medical University, Kaohsiung 807, Taiwan; beautyhappy19@gmail.com (S.-H.C.); golden3p@gmail.com (Y.-W.C.); 3 Research Center for Environmental Medicine, Kaohsiung Medical University, Kaohsiung 807, Taiwan; 4 School of Medical and Health Sciences, Fooyin University, Kaohsiung 831, Taiwan; XX377@fy.edu.tw; 5 Department of Biological Science and Technology, College of Biological Science and Technology, National Chiao Tung University, Hsinchu 300, Taiwan

**Keywords:** pheophytin-b, nitric oxide, prostaglandin E2, cytokine, macrophages, lipopolysaccharide

## Abstract

Sepsis is an overwhelming systemic response to infection that frequently results in tissue damage, organ failure, and even death. Nitric oxide (NO), prostaglandin E2 (PGE2), and cytokine overproduction are thought to be associated with the immunostimulatory cascade in sepsis. In the present study, we analyzed the anti-inflammatory efficacy of the pheophytin-b on both RAW 264.7 murine macrophage and purified human CD14^+^ monocytes stimulated with lipopolysaccharide (LPS) and elucidated the mechanisms by analyzing the cell signaling pathways known to be activated in sepsis. Pheophytin-b suppressed the overexpression of NO, PGE2, and cytokines in LPS-stimulated macrophages without inducing cytotoxicity. It also reduced NOS2 and COX-2 mRNA and protein levels. The inhibitory effects on NO, PGE2, and cytokine overproduction arose from the suppression of STAT-1 and PI3K/Akt pathways; no changes in NF-κB, MAPK, and AP-1 signaling were detected. Thus, pheophytin-b may represent a potential candidate to beneficially modulate the inflammatory response in sepsis.

## 1. Introduction

Sepsis, severe sepsis, and septic shock remain to be the main causes of death among critically ill patients [1], and the number of new sepsis cases are increasing [2,3] despite advances in understanding its etiology and the development of new therapeutic strategies [4]. The management of severe sepsis is especially challenging because of its high mortality rate [5]. Despite improvements in the quality of critical care, the mortality rate of sepsis remains high, ranging from 18% to 50% [1,4,6]. Although several immunomodulation and anticoagulation drugs have been developed to treat sepsis, their efficacies are limited, and some have been withdrawn from the market [3]. Therefore, the development of new agents that could be applied for the treatment of sepsis is critical [7,8].

Microbial components are always potential stimulators for macrophage activation. Macrophage is the major effector of innate immune response, which associates with cytokines secretion, phagocytosis, and antigen presenting. Sepsis is known to be correlated with the imbalance between pro-inflammatory and anti-inflammatory [9]. Patients with sepsis exhibit heightened inflammatory responses, including nitric oxide (NO) production, cytokines secretion, and prostaglandin synthesis [10], which are thought to be induced at least in part by the endotoxin lipopolysaccharide (LPS) a major component of the cell walls of many Gram-negative sepsis-inducing microbes. LPS is demonstrated to influence thousands of cytokine-related genes expression in macrophage [11]. Additionally, excessive NO production during sepsis, which is mainly generated by NO synthase 2 (NOS2, also named inducible NO synthase) encoded by *NOS2* gene [12], has been suggested to be one of the main factors leading to tissue injury induced by septic shock [13]. NO and overexpressed cytokines, such as tumor necrosis factor-α (TNF-α), interleukin-1β (IL-1β), and IL-6, were also shown to cause sepsis-related systemic inflammation [14] and myocardial depression in sepsis and septic shock [15,16,17]. Prostaglandin E2 (PGE2) is primarily synthesized by cyclooxygenase-2 (COX-2), which is also responsible for sepsis-related inflammatory symptoms and signs [18]. COX-2 can be overexpressed following stimulation with LPS [19].

We have previously found that pheophytin-a, which is a chlorophyll-related compound extracted from green tea, elicits anti-inflammatory effects [20]. Pheophytin-b, another chlorophyll-related compound, possesses medically beneficial properties, such as anti-tumor effects [21], anti-genotoxic effects [22], and anti-oxidative activity [23]. Pheophytin-a and pheophytin-b show the difference in the chemical structure C-7, where there is a methyl group in pheophytin-a and a formyl group in pheophytin-b. However, the precise role of pheophytin-b in sepsis-related inflammation remains unknown. Therefore, in this study, we investigated the efficacy of pheophytin-b using the RAW 264.7 murine cell model as well as purified human CD14^+^ monocytes. Furthermore, we elucidated the molecular mechanisms by which pheophytin-b exerts its effects.

## 2. Results

### 2.1. Pheophytin-b Does Not Induce Macrophage Cytotoxicity

**Figure 1:**
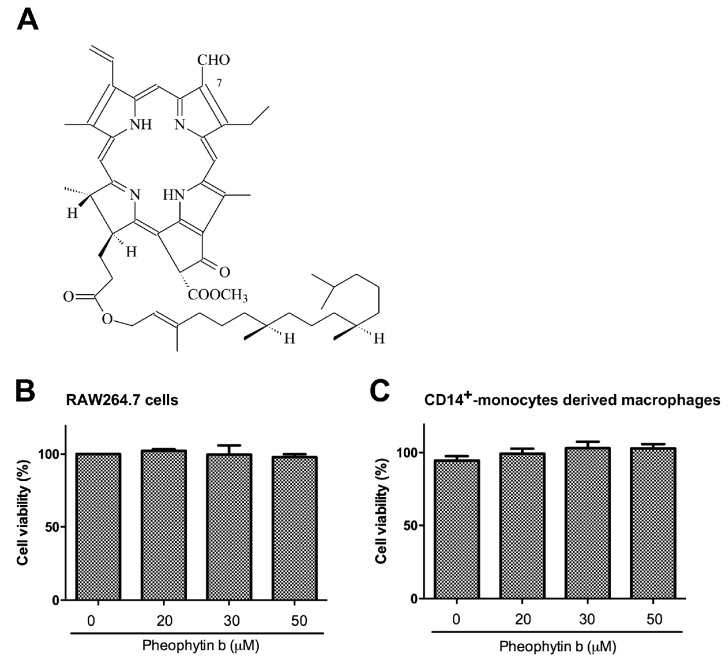
Pheophytin-b treatment had no significant effect on the viabilities of RAW 264.7 cells or human CD14^+^ monocyte-derived macrophages. (**A**) Chemical structure of pheophytin-b. Both RAW 264.7 cells (**B**) and human CD14^+^ monocyte-derived macrophages (**C**) were treated with pheophytin-b for 24 h at the indicated concentrations and viabilities were measured using an Alamar Blue assay. Data are expressed as the means ± standard deviations (SD) of five independent experiments (*n* = 5).

The chemical structure of pheophytin-b is illustrated in Figure 1A. As shown in Figure 1B, pheophytin-b did not influence the cell viability of RW264.7 cells in doses up to 50 μM. Similarly, analysis of human CD14^+^ monocyte-derived macrophages showed no significant change in macrophage viability upon treatment with pheophytin-b at doses up to 50 μM (Figure 1C; the purity of human CD14^+^ monocyte has been shown in Figure S1). Thus, all subsequent experiments used in the present study were performed using a maximal 50 μM dose of pheophytin-b.

### 2.2. Effects of Pheophytin-b on NO and PGE2 Production in LPS-Stimulated Macrophages

**Figure 2:**
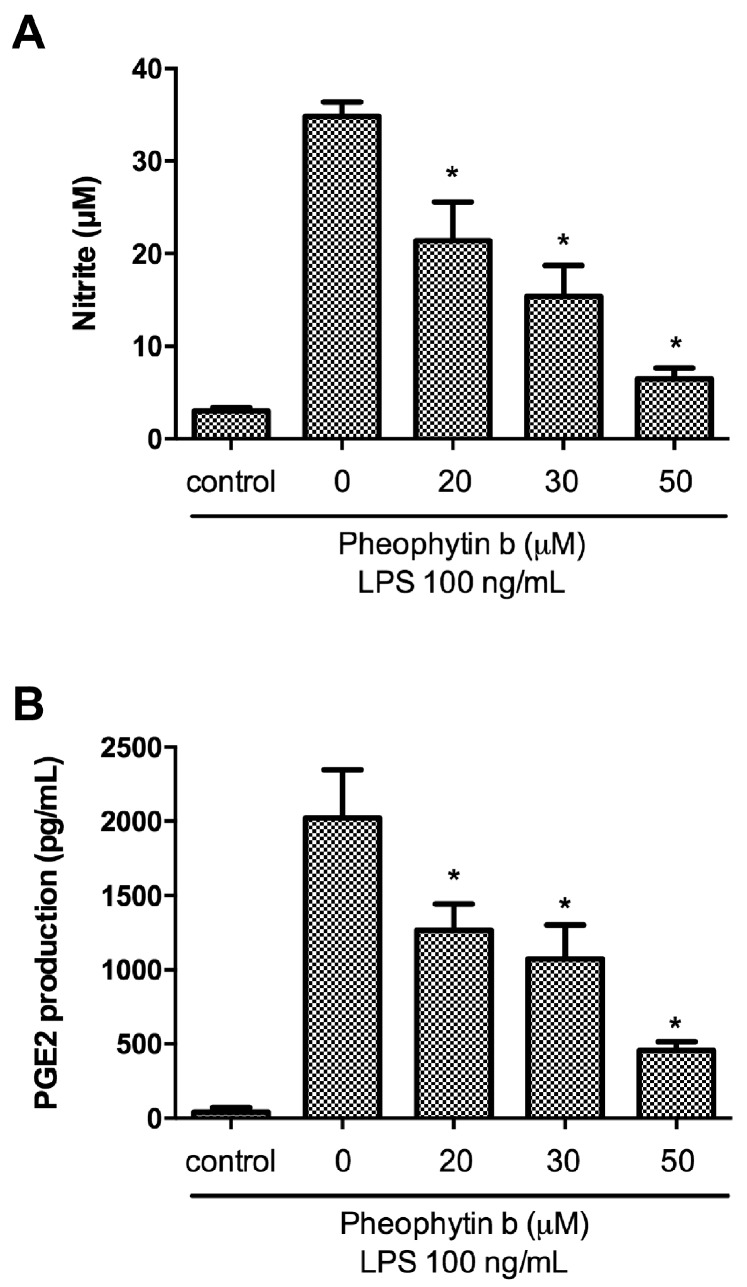
The pre-treatment with pheophytin-b exerted significant repression of LPS-induced NO and PGE2 production. RAW 264.7 cells were pre-treated with the indicated concentrations of pheophytin-b for 30 min, and the production of nitrite (**A**) and PGE2 (**B**) were quantified after 6 h and 16 h of LPS (100 ng/mL) stimulation, respectively. * *p* < 0.05 vs. LPS-treated cells (by Student’s *t*-test, *n* = 3).

In the present study, pre-treatment of RAW 264.7 cells with pheophytin-b for 30 min elicited significant, dose-dependent suppression of LPS-induced NO production as detected by a reduction in nitrite levels (*p* < 0.05; Figure 2A). Furthermore, using an enzyme-linked immunosorbent assay (ELISA), pre-treatment with pheophytin-b significantly attenuated LPS-stimulated PGE2 production by RAW 264.7 cells also in a dose-dependent manner (*p* < 0.05; Figure 2B).

### 2.3. Effects of Pheophytin-b on NOS2 and COX-2 Expression

**Figure 3:**
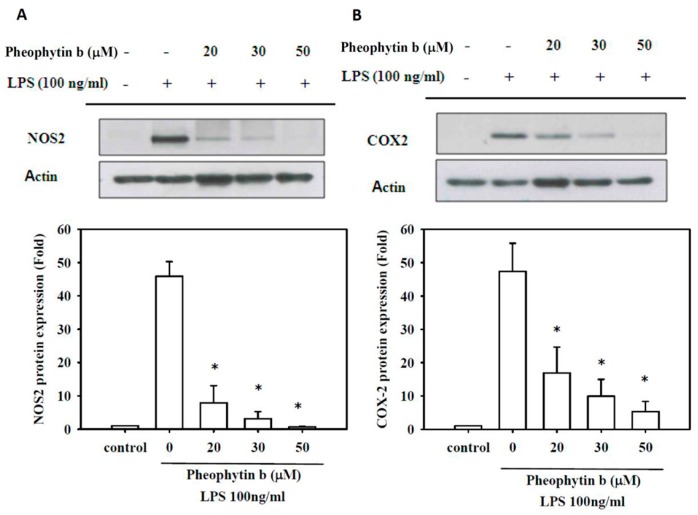
Pheophytin-b suppressed NOS2 and COX-2 protein synthesis in LPS-stimulated RAW 264.7 cells. Cells were pre-treated with the indicated concentrations of pheophytin-b for 30 min and stimulated with LPS for 6 h. NOS2 (**A**) and COX-2 (**B**) protein levels were determined by Western blot analysis, quantified, and normalized to those of the control group. Bottom panels show the fold-change compared to the control group (without LPS stimulation). Actin levels were analyzed as a control to ensure equal loading. * *p* < 0.05 vs. LPS-treated cells (by Student’s *t*-test, *n* = 3).

**Figure 4:**
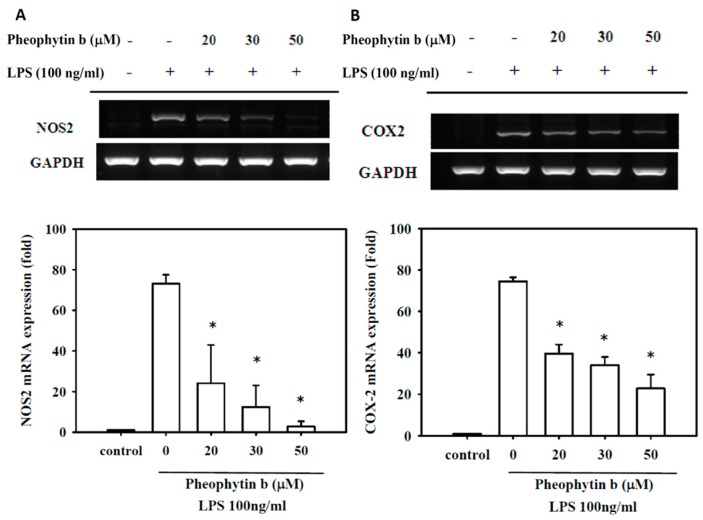
Pheophytin-b suppressed NOS2 and COX-2 mRNA expression levels in LPS-stimulated RAW 264.7 cells. Cells were pre-treated with the indicated concentrations of pheophytin-b for 30 min and stimulated with LPS for 3 h. NOS2 (**A**) and COX-2 (**B**) mRNA levels were determined by QRT-PCR analysis, quantified, and normalized to those of the control group. Bottom panels show the fold-change compared to the control group (without LPS stimulation). Expression of GAPDH was analyzed as a control to ensure equal loading. * *p* < 0.05 vs. the cells treated with LPS alone (by Student’s *t*-test, *n* = 3).

LPS-induced expression of NOS2 protein and mRNA were also repressed in RAW 264.7 cells with pheophytin-b in a dose-dependent manner (*p* < 0.05; Figure 3A and Figure 4A, respectively). Similarly, pre-treatment with pheophytin-b attenuated *COX-2* gene expression in a dose-dependent manner, as indicated by decreased protein synthesis and mRNA levels (*p* < 0.05; Figure 3B and Figure 4B, respectively).

### 2.4. Effects of Pheophytin-b on Cytokine Production in LPS-Stimulated Macrophages

**Figure 5:**
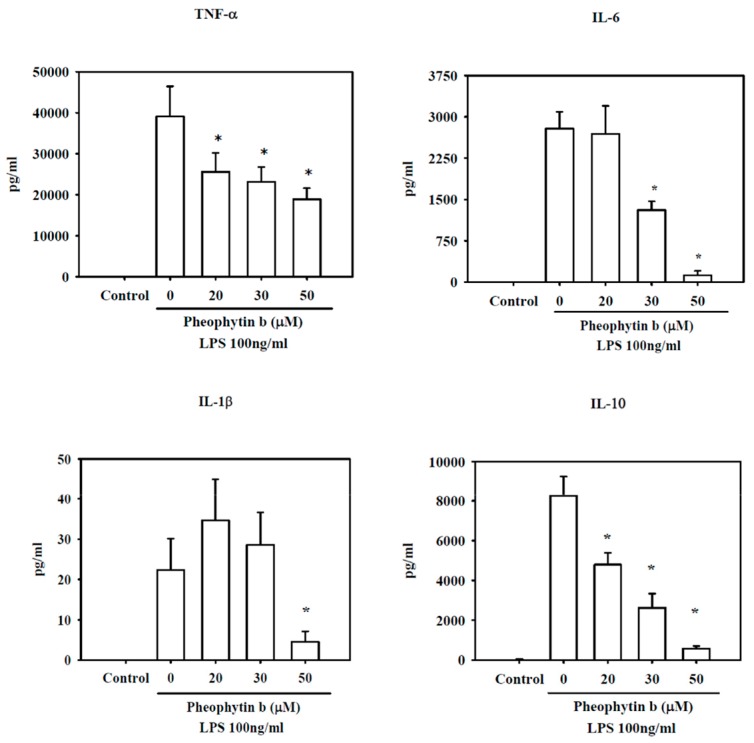
Pheophytin-b ameliorates cytokine expression induced by LPS in RAW 264.7 cells. RAW 264.7 cells were pre-treated with the indicated concentrations of pheophytin-b for 30 min followed by LPS (100 ng/mL) treatment for 24 h. TNF-α, IL-1β, IL-6, and IL-10 concentrations were measured in the culture supernatants by ELISA. Results show the means ± SEM of three experiments. * *p* < 0.05 vs. cells treated with LPS alone (by Student’s *t*-test, *n* = 3).

We also analyzed the effects of pheophytin-b on cytokine production in LPS-stimulated RAW 264.7 cells. As shown in Figure 5, LPS increased TNF-α, IL-6, IL-1β, and IL-10 levels as compared to untreated controls; however, all of these cytokines were significantly decreased with 50 µM pheophytin-b treatment (all *p* < 0.05).

### 2.5. Effects of Pheophytin-b on PGE2 and Cytokine Production in CD14^+^ Monocyte-Derived Macrophages after LPS Stimulation

**Figure 6:**
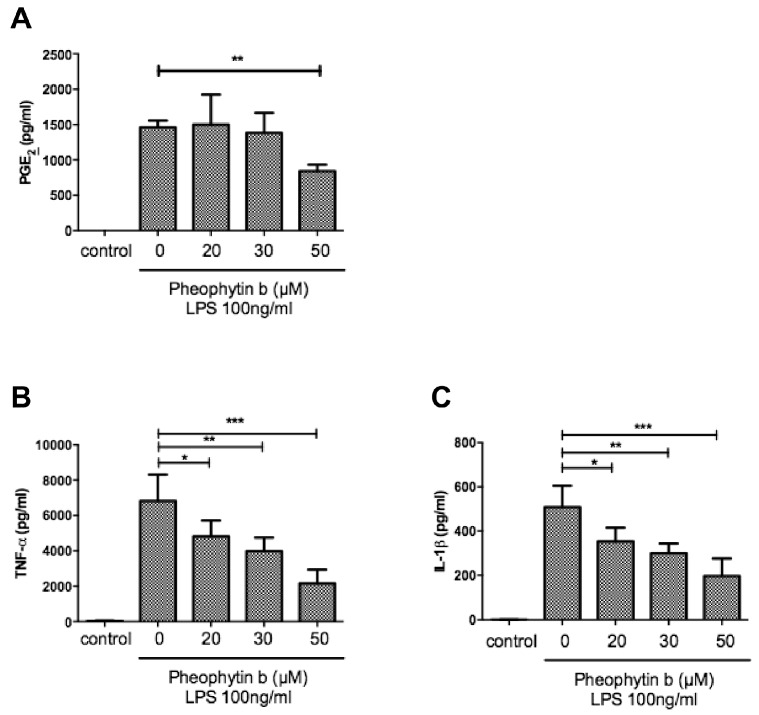

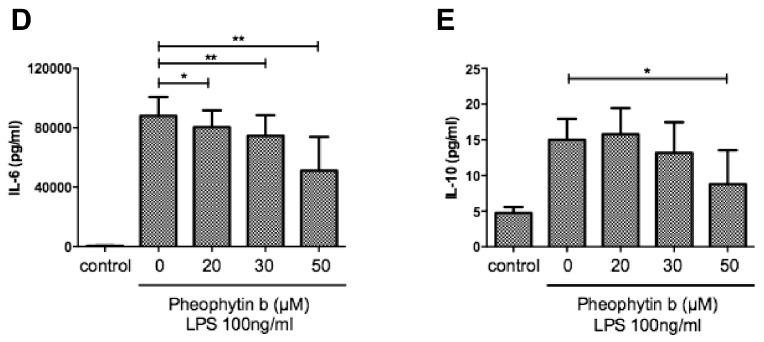
Effects of pheophytin-b on LPS-induced PGE2 and cytokine expression profiles by human CD14^+^ monocyte-derived macrophages. Human CD14^+^ monocyte-derived macrophages were pre-treated with the indicated concentrations of pheophytin-b for 30 min followed by LPS (100 ng/mL) for 24 h. The secretions of PGE2 (**A**), TNF-α (**B**), IL-1β (**C**), IL-6 (**D**), and IL-10 (**E**) from LPS-induced cells pre-treated with/without pheophytin-b were measured. Results show the means ± standard deviation of three experiments. * *p* < 0.05, ** *p* < 0.01, *** *p* < 0.001 vs. cells treated with LPS alone (Student’s *t*-test, *n* = 3).

To confirm the anti-inflammatory effects of pheophytin-b on macrophages, we also used CD14^+^ monocyte-derived macrophages to perform ex vivo experiments. As is shown in Figure 6, LPS-induced PGE2 and inflammatory cytokines were all suppressed by pheophytin-b. However, NO production in the supernatants of LPS-stimulated CD14^+^ monocyte-derived macrophages was near the limit of detection (data not shown) [24].

### 2.6. Effects of Pheophytin-b on LPS-Mediated Signal Transduction Pathways in Macrophages

We next investigated the regulatory mechanism and pathway responsible for the suppression of inflammation in LPS-stimulated RAW 264.7 cells by first examining the expression of transcription factors and activation of signal transduction pathways. NF-κB has an important role in the induction of inflammation [25,26]; therefore, we examined the nuclear translocation of phosphorylated-p65 (p-p65) and cytoplasmic IkBα. As shown in Figure S2, there was no significant change in p-p65 translocation or IkBα expression in response to pheophytin-b treatment in LPS-stimulated RAW 264.7 cells.

**Figure 7:**
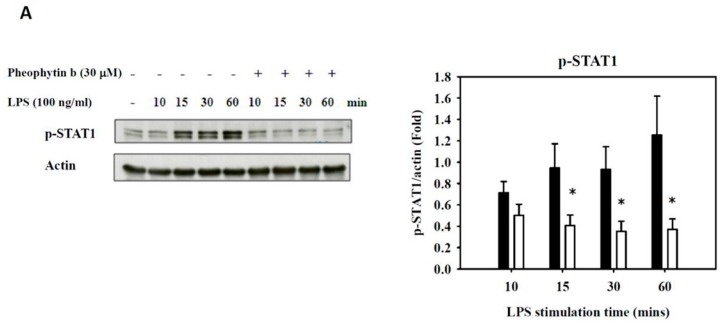

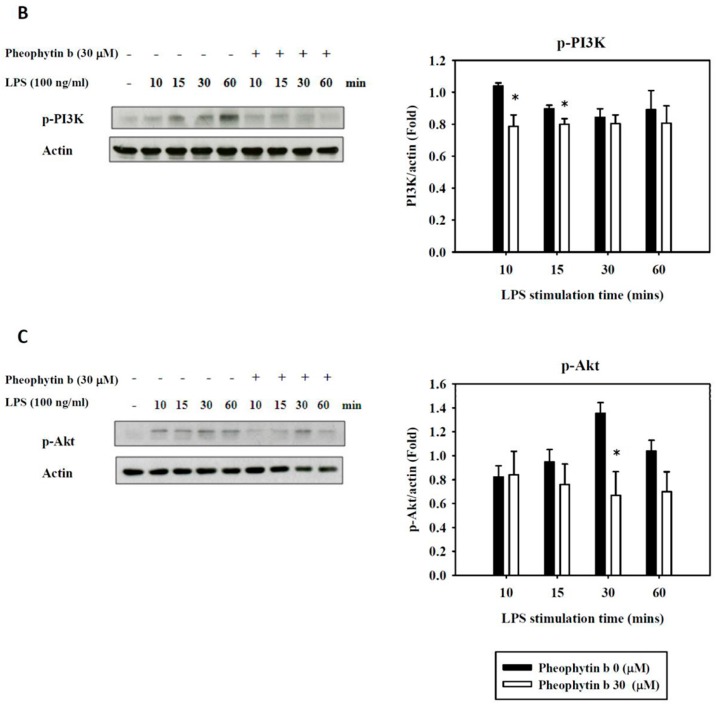
Effects of pheophytin-b on the STAT1 and PI3K/Akt pathways in LPS-stimulated RAW 264.7 cells. Western blot analysis of STAT1 (**A**), PI3K (**B**), and Akt (**C**) phosphorylation induced by LPS (100 ng/mL) was significantly suppressed by pheophytin-b. Cells were pre-treated with 30 μM pheophytin-b for 30 min, and total protein was harvested at four different time points (10, 15, 30 and 60 min) after LPS stimulation (100 ng/mL). * *p* < 0.05 vs. cells treated with LPS alone (Student’s *t*-test, *n* = 3).

Another transcription factor that might bind to the *NOS2* promoter is AP-1 [27], which related pathway includes c-Jun and c-Fos [28]. In addition, several signal transduction pathways, such as MAPKs and Akt, have also been demonstrated to be involved in regulating the sepsis-induced production of NO and pro-inflammatory cytokines and chemokines [14,29,30]. Hence, we investigated the activity of several MAPK pathway members, including phosphorylated p38, ERK1/2, and JNK; however, pheophytin-b had no significant inhibitory effect on these pathways (Figure S3). There were also no significant effects of pheophytin-b on the c-Fos and c-Jun pathways in LPS-stimulated RAW 264.7 cells (Figure S4). In contrast, 30 μM pheophytin-b significantly suppressed the levels of phosphorylated STAT1 (p-STAT1), PI3K (p-PI3K), and Akt (p-Akt), which stimulated by LPS (Figure 7).

## 3. Discussion

Sepsis is a lethal host response to a microbial infection, and its high mortality rate is related to a dysregulation of inflammatory mediators. Therefore, a possible therapeutic approach to sepsis treatment may focus on anti-inflammatory agents to suppress the proinflammatory cytokine storm. The importance of natural products for disease treatment and drug discovery was again recently emphasized and highlighted in the 2015 Nobel Prize in Medicine [31]. We previously reported that the chlorophyll-related compound, pheophytin-a, suppresses LPS-induced NOS-2, PGE2, and IL-1β production by macrophages [20]. Here, in this study, we present evidence that pheophytin-b also possesses similar anti-inflammatory effects on LPS-stimulated murine macrophages (RAW264.7) and human CD14^+^ monocyte-derived macrophages without inducing cytotoxicity. Both NO and PGE2 end products were reduced with pheophytin-b, in addition to both mRNA and protein of the NOS-2 and COX-2 in a dose-dependent manner.

Compared to pheophytin-a, pheophytin-b is more tolerable to cells [20]. Specifically, RAW 264.7 cell proliferation was significantly reduced when the dose of pheophytin-a was titrated up to 25 μM [20]. In contrast, even the treatment concentrations of pheophytin-b were increased up to 50 μM, there were no significant cytotoxicity effects on both the RAW 264.7 cells and the human CD14^+^ monocyte-derived macrophages. These results suggested that pheophytin-b may have a larger “therapeutic range” than pheophytin-a. The differential cytotoxicities observed between pheophytin-a and pheophytin-b may be mediated by diverging cell signaling regulation. Although both molecules suppressed the STAT-1 pathway without influencing NF-κB or AP-1 signaling [20], pheophytin-a specifically increased ERK1/2 activity. In contrast, no changes in MAPK signaling (phosphorylated p38, ERK1/2, and JNK) were observed with pheophytin-b. Further studies are required to examine the specific cellular pathways underlying pheophytin-a-induced cytotoxicity.

Unlike the anti-inflammatory activity observed for the edible brown alga *Saccharina japonica* and its constituents [32], pheophytin-b decreased COX-2 expression in both RAW 264.7 cells and CD14^+^ monocyte-derived macrophages. Our findings also suggest that pheophytin-b significantly suppresses NOS-2 expression at both the transcriptional and translational levels in LPS-stimulated RAW 264.7 cells. Although NO production from human CD14^+^ monocyte-derived macrophages is difficult to detect [24,33], we did discern a suppression of nitrite production with pheophytin-b. Moreover, the inhibitory effect of pheophytin-b on the inflammatory response was evident in the significant suppression of LPS-induced PGE2, TNF-α, IL-1β, IL-6, and IL-10 by macrophages.

IL-10, which is thought to be an anti-inflammatory cytokine, was also suppressed by pheophytin-b, which is similar to that observed for other compounds, including synthetic chalcone and flavone derivatives, wogonin, and phenylpropanoid dimers isolated from *Nectandra leucantha* [34,35,36,37,38]. A possible explanation is that, when pheophytin-b inhibits inflammation, the subsequent production of an anti-inflammatory cytokine, such as IL-10, can be lessened to a degree that corresponds to the decrease in inflammatory cytokine production.

**Figure 8:**
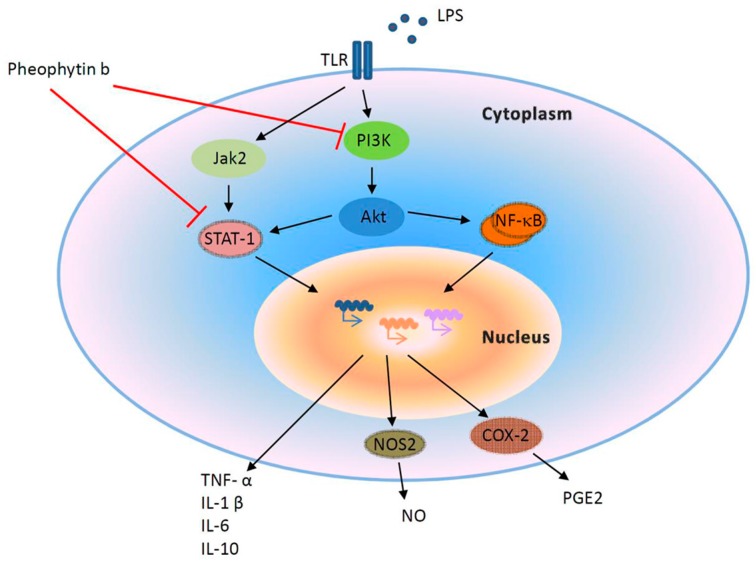
Proposed mechanism depicting the effect of pheophytin-b on LPS-stimulated macrophages. Production of NO, PGE2, IL-1β, IL-6, IL-10, and TNF-α induced by LPS are suppressed by pheophytin-b inhibition of STAT-1 and PI3K/Akt signaling pathways.

Inhibition of pathophysiological NO has been suggested to be a therapeutic target in sepsis although several clinical studies have been prematurely terminated due to the absence of therapeutic benefits [13]. The promoter of the *NOS2* gene, which is responsible for excessive NO production in sepsis in response to LPS [12], can be bound by several transcriptional factors, such as NF-κB, AP-1, and STAT-1 [39,40,41]. In addition, the PI3K and Akt pathways also play an important role in the production of NO under LPS simulation [42]. In the present study, pheophytin-b exerted its anti-inflammatory effect via PI3K, Akt, and the subsequent STAT-1 pathway. However, no activation of the NF-κB or MAPK pathways was observed with pheophytin-b. Figure 8 illustrates our proposed scheme depicting the immune-modulatory mechanism of pheophytin-b. In a mechanism similar to that reported for 6-dehydrogingerdione, a component of ginger [43], as well as the flavonoids, phloretin and phorizin [14], pheophytin-b suppressed LPS-induced STAT1, PI3K, and Akt activation, resulting in a reduction in cytokines, NO, and PGE2 production.

In conclusion, pheophytin-b has potent anti-inflammatory effects in LPS-stimulated macrophages. Therefore, this compound could represent a potential agent for use as an immunomodulatory drug for patients with sepsis. In subsequent experiments, pheophytin-b has shown rare activity that would interfere with the phagocytosis of human macrophages (data not shown). Further in vivo analyses are required to fully examine the impact of pheophytin-b on sepsis.

## 4. Materials and Methods

### 4.1. Reagents and Antibodies

Pheophytin-b was purchased from Wako Pure Chemical Industries, Ltd., Osaka, Japan. LPS (*Escherichia coli* O26:B6, L2654) was purchased from Sigma (St. Louis, MO, USA).

The NOS2 antibody was purchased from BD Biosciences (San Jose, CA, USA). Antibodies against p44/p42 MAP Kinase (Thr202/Tyr204), p38 MAP Kinase (Thr180/Tyr182), SAPK/JNK (Thr183/Tyr185), ERK1/2 MAP Kinase (Thr202/Tyr204), phospho-IκB-α (Ser32/36), STAT1 (Tyr701), Akt (Ser473), PI3K p85 (Tyr458)/p55 (Tyr199), c-Fos (9F6), c-Jun (Ser73), and COX-2 were all purchased from Cell Signaling (Danvers, MA, USA). The NF-κB p65 rabbit monoclonal antibody (Cell Signaling), lamin B antibody (Santa Cruz, CA, USA), and mouse monoclonal beta-Actin antibody (Abcam, Cambridge, MA, USA) were also utilized.

### 4.2. RAW 264.7 Cell Culture

RAW 264.7 cells, derived from murine macrophages, were purchased from the Bioresource Collection and Research Center (BCRC, Hsinchu, Taiwan). RAW 264.7 cells were cultured in DMEM (GIBCO, Carlsbad, CA, USA) with 10% heat-inactivated fetal bovine serum (Biological Industries, Cromwell, CT, USA), 100 U/mL penicillin, 100 μg/mL streptomycin, 25 μg/mL amphotericin B (Biological Industries), and 2 mM l-glutamine (GIBCO). The cells were culture in a 37 °C incubator with 5% CO_2_.

### 4.3. Ex Vivo Isolation of Human CD14^+^ Monocytes and Stimulation into Macrophages

This project has been approved by the Institutional Review Board of Kaohsiung Medical University Hospital (KMUH-IRB-20140303). Human CD14^+^ monocytes were isolated from human peripheral blood mononuclear cells (PBMCs) obtained from blood buffy coats according to the methods previously described [44,45]. In brief, 20 mL of each blood sample was collected into aseptic tubes containing EDTA from the veins of three healthy volunteers for independent experiments. PBMCs were isolated by gradient centrifugation on Ficoll–Hypaque (GE Healthcare, Wauwatosa, WI, USA) with anti-CD14 microbeads (Miltenyi Biotec GmbH, Bergisch Gladbach, Germany), according to the manufacturer’s instructions. Cells were washed twice with PBS and resuspended in RPMI 1640 medium (Mediatech, Manassas, VA, USA) supplemented with 10% FBS and antibiotics (100 U/mL penicillin, 100 μg/mL streptomycin, and 0.25 g/mL Amphotericin B). For purity examination, CD14^+^ cells were labeled with CD14-FITC and CD3-PE and analyzed by flow cytometry. PBMCs were used as a control. CD14^+^ positive cells showed >95% of purity (refer to Figure S1 and see below). We then stimulated the purified human CD14^+^ monocytes to differentiate into macrophages by adding human GM-CSF (10 ng/mL) (PeproTech, Rocky Hill, NJ, USA) for 6 days at 37 °C in 5% CO_2_ [46] after which they were prepared for subsequent pheophytin-b treatment.

### 4.4. Cytotoxicity Analysis Following Pheophytin-b Treatment

Cytotoxicity analyses following stimulation with pheophytin-b were evaluated in both types of macrophages using an Alamar Blue Cell viability kit (Serotec Ltd. Scandinavia, Hamar, Norway). In brief, 200 μL of 10% Alamar Blue reagent was added into a 96-well plate, containing 2 × 10^4^ cells/well and 200 μL of medium, the cells of which were treated with 0, 20, 30, and 50 μM pheophytin-b for 24 h. Colorimetric analyses were obtained with an ELISA plate reader at 570 and 600 nm, and the cytotoxicity of pheophytin-b treatment was evaluated relative to controls.

### 4.5. Western Blot and QRT-PCR Analysis of NO2 and COX-2 Protein in Pheophytin-b Treatment and LPS Stimulation of RAW 264.7 Cells

RAW 264.7 cells were treated with the indicated concentrations of pheophytin-b. After 30 min, the cells were stimulated with LPS (100 ng/mL). After 3 h, the cells were collected and prepared for real-time quantitative PCR (QRT-PCR) analysis [20,47]. After 6 h, the cells were collected to measure NOS2 and COX-2 protein production by Western blot analysis. Expression of actin was analyzed as a control to ensure equal loading. For QRT-PCR, the mRNA was extracted from RAW 264.7 cells using the RNAspin Mini RNA Isolation Kit (GE Healthcare, Buckinghamshire, UK). QRT-PCR analysis measuring NOS2 and COX-2 mRNA levels was then performed in a Roche LightCycler (Mannheim, Germany), based on a method previously described [43]. Primers used for PCR were as follows: NOS2 forward primer, 5′-CCCTTCCGAAGTTTCTGGCAGCAGC-3′ and reverse primer, 5′-GGCTGTCAGAGAGCCTCGTGGCTTTGG-3′; COX-2 forward primer, 5′-CATTGATGGTGGCTGTTTTG-3′ and reverse primer, 5′-GTTGCTGGGGGAAGAAATGT-3′; GAPDH forward primer, 5′-TCCACCACCCTGTTGCTGTA-3′ and reverse primer, 5′-ACCACAGTCCATGCCATCAC-3′.

### 4.6. Measurement of Nitrite Release Indicative of NO Production

The concentration of nitrite, a stable metabolite of NO, in the supernatant has been measured as an indicator of NO production [39] using the Griess reagent system (Promega Biotech Co., Ltd., Madison, WI, USA) according to the manufacturer’s instructions. In brief, cells were treated with various doses of pheophytin-b and LPS after which 50 μL of each supernatant was carefully transferred into a 96-well plate, and 50 µL of Griess reagent was subsequently added. In the Griess reagent system, nitrite standard (0.1 M sodium nitrite) was prepared for a reference curve from the concentration of 100 µM to that of 1.56 µM. After color development for 10 min, absorbance was measured at a wavelength of 520 nm. This absorbance was then normalized using the standard curve to obtain the nitrite concentration.

### 4.7. Transcription Factor (NF-κB and AP-1) Translocation Assays

Cytosolic and nuclear extracts were isolated from RAW 264.7 cells according to a previously described protocol [43]. In brief, cytosolic and nuclear proteins were harvested 10, 15, 30, and 60 min after LPS stimulation, based on the method previously described [20].

### 4.8. Enzyme-Linked Immunosorbent Assay (ELISA) Analyses

RAW 264.7 cells were plated at a density of 4 × 10^5^ cells per well in 12-well plates. After the cells were pre-treated with different concentrations of pheophytin-b, they were treated with LPS, and the supernatants were collected after 16 h for detection of PGE2 or 24 h for cytokines. PGE2 protein concentrations were measured using ELISA kits for PGE2 (R&D Systems, Minneapolis, MN, USA); TNF-α, IL-1β, IL-6, and IL-10 levels were measured using ELISA kits from eBioscience (San Diego, CA, USA). Each treatment was performed in duplicate wells, and at least three parallel experiments were performed.

### 4.9. Signal Transduction Pathway Analysis

Nuclear translocation of components of the NF-κB pathway (IkBα and p65) and phosphorylation of the MAPK pathway (i.e., p38, ERK1/2, and JNK), c-Fos, c-Jun, STAT-1, PI3K, and Akt proteins were analyzed in RAW 264.7 cells pretreated with pheophytin-b for 30 min followed by induction with LPS (100 ng/mL). Total and nuclear proteins were harvested at 10, 15, 30, and 60 min after stimulation with LPS. Each experiment was repeated three times.

### 4.10. Statistical Analysis

Experimental results are expressed as the means ± standard errors of mean (SEM), unless otherwise specified as standard deviations (SD), with *n* indicating the number of experiments. Statistical significance was determined by Student’s *t*-test. All differences were considered significant at a *p*-value of <0.05.

## Supplementary Materials

The following are available online at www.mdpi.com/1422-0067/18/12/2637/s1.

## Abbreviations

COX-2	cyclooxygenase-2
ELISA	enzyme-linked immunosorbent assay
IL	interleukin
LPS	lipopolysaccharide
NO	nitric oxide
NOS2	NO synthase 2
PGE2	prostaglandin E2
QRT-PCR	real-time quantitative PCR
SD	standard deviations
SEM	standard errors of mean
TNF-α	tumor necrosis factor-α
