# Supplementary material for: Lipopolysaccharide-Induced Nitric Oxide, Prostaglandin E2, and Cytokine Production of Mouse and Human Macrophages Are Suppressed by Pheophytin-b
Source: Int J Mol Sci. 2017 Dec 6;18(12):2637. doi: 10.3390/ijms18122637 (PMC5751240; doi:10.3390/ijms18122637)
Supplement: Supplementary file 1 [file ijms-18-02637-s001.pdf]

Supplementary materials

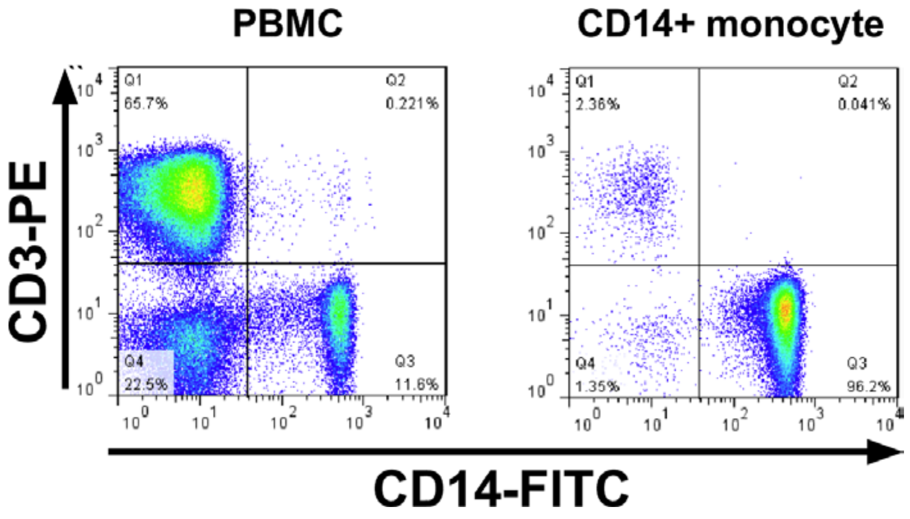

Figure S1. The purity of CD14⁺ monocytes was evaluated by flow cytometry. CD14⁺ monocytes were enriched and isolated from PBMCs using the MiniMACS® Separator and human CD14 MicroBeads kit, according to the manufacturer's instructions. Post-enrichment, CD14⁺ cells were labeled with CD14-FITC and CD3-PE and analyzed by flow cytometry. PBMCs were used as a control. After enrichment, CD14⁺ positive cells due to >95% of purity of monocytes was noted.

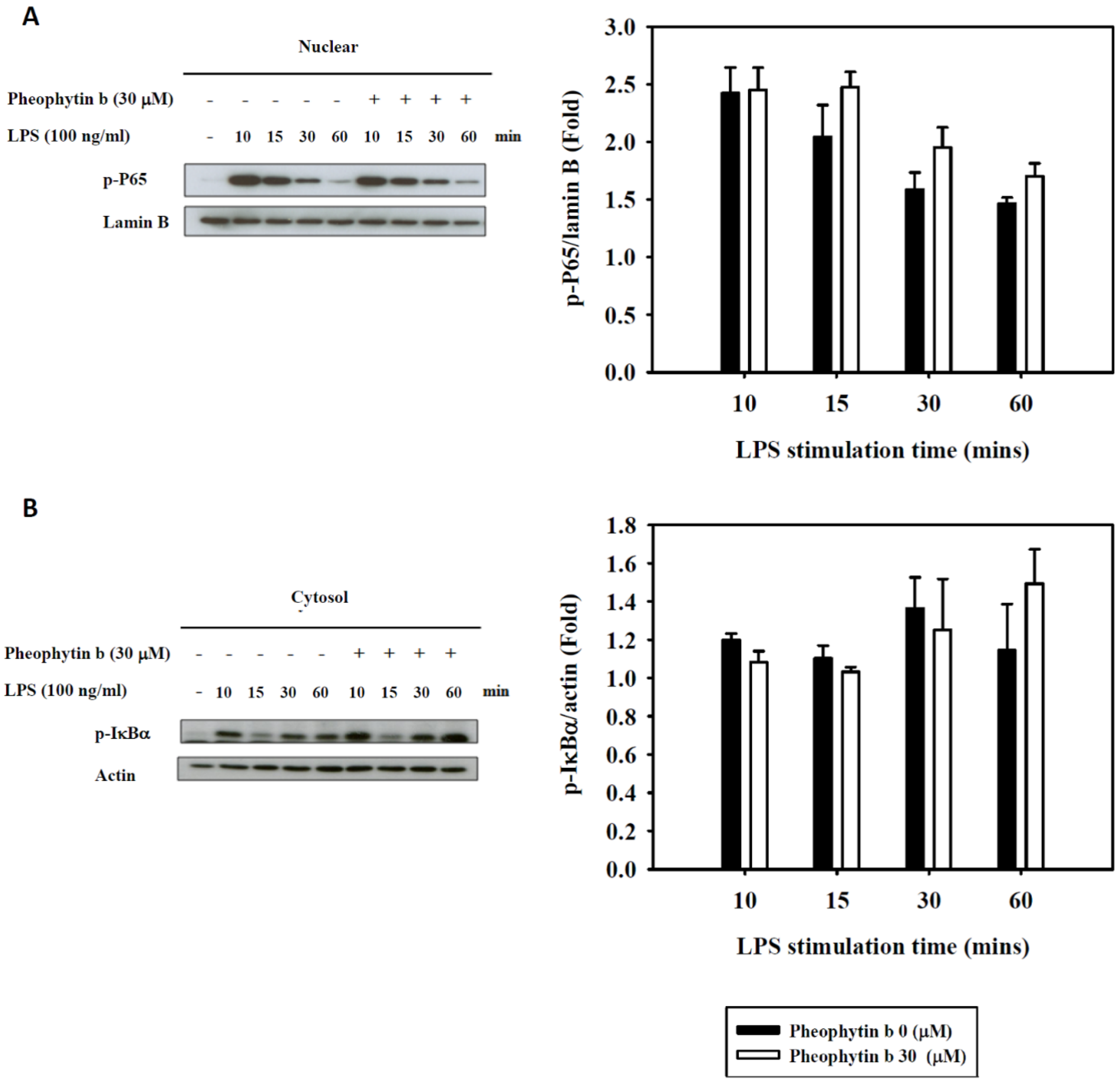

Figure S2. There were no significant effects of Pheophytin-b (30μM) on the NF-κB pathway in LPS-stimulated RAW 264.7 cells. Cells were pre-treated with or without 30μM Pheophytin-b for 30 min, and nuclear and cytosolic proteins were harvested at four indicated time points (10, 15, 30 and 60 min) after LPS (100 ng/mL) stimulation. Western blot analysis of phosphorylated p65 levels (**A**) in the nuclear fraction of LPS-stimulated RAW 264.7 cells and its semi-quantification after normalization to lamin B levels in three different experiments. Western blot analysis of phosphorylated IκBα expression (**B**) in the cytosolic fraction of LPS-stimulated RAW 264.7 cells and its semi-quantification after normalization to actin levels in three different experiments.

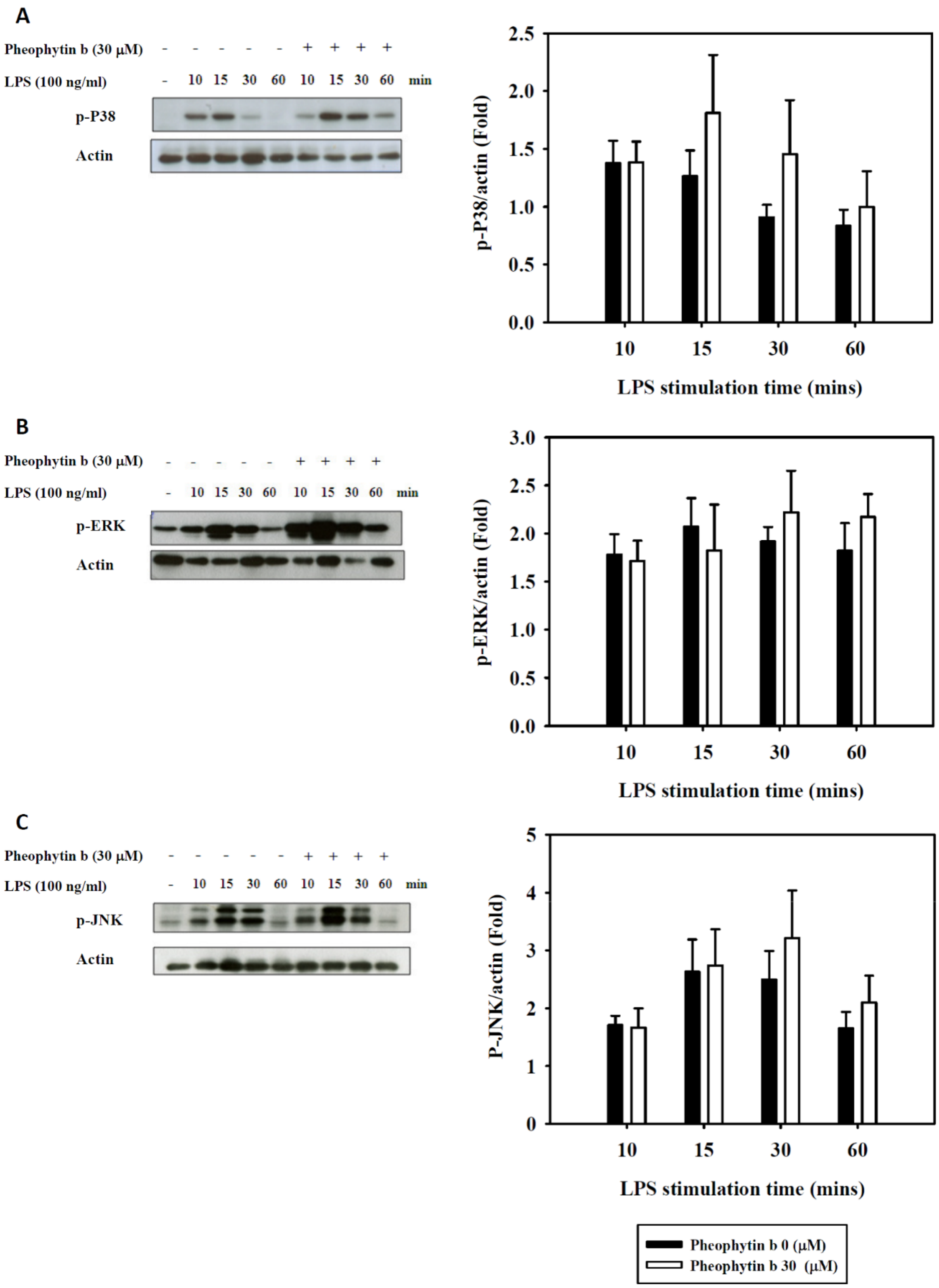

Figure S3. There were no significant effects of Pheophytin-b (30μM) on the MAPK signaling pathways in LPS-stimulated RAW 264.7 cells. Cells were pre-treated with or without 30μM Pheophytin-b for 30 min, and total protein was harvested at four indicated time points (10, 15, 30 and 60 min) after LPS (100 ng/mL) stimulation. Western blot analysis of phosphorylated p38 levels (**A**), phosphorylated ERK levels (**B**), and phosphorylated JNK expression (**C**) in LPS-stimulated cells and its semi-quantification after normalization to actin in three different experiments.

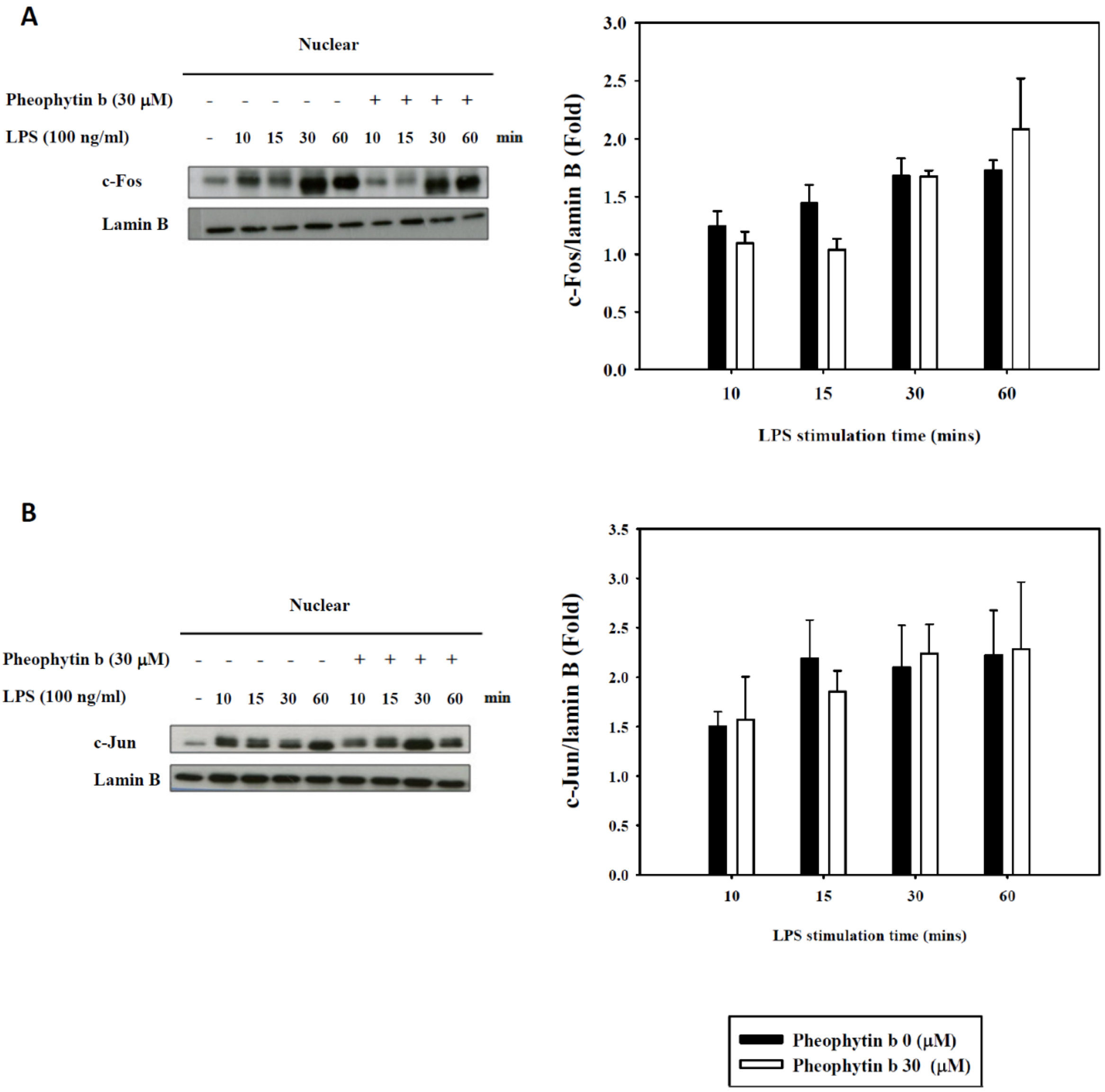

Figure S4. There were no significant effects of Pheophytin-b (30μM) on the c-Fos and c-Jun pathways in LPS-stimulated RAW 264.7 cells. Cells were pre-treated with or without 30μM Pheophytin-b for 30 min, and nuclear extracts were harvested at four indicated time points (10, 15, 30 and 60 min) after LPS (100 ng/mL) stimulation. Western blot analysis of c-Fos expression (**A**) and c-Jun expression (**B**) in the nuclear fraction of LPS-stimulated RAW 264.7 cells and its semi-quantification after normalization to lamin B in three different experiments.
